# Address: Introductory to the Fifth Course of Lectures in the Atlanta Medical College

**Published:** 1859-07

**Authors:** Jos. P. Logan

**Affiliations:** Atlanta, Ga.


ATLANTA

Medical and Surgical Journal.

VOL. IV.] JULY, 1859. [No. XI.

### ORIGINAL COMMUNICATIONS.

### ARTICLE I.

*Address: Introductory to the Fifth Course of Lectures in the Atlanta Medical College.* By Prof. JOS. P. LOGAN, M. D., Atlanta, Ga.

GENTLEMEN OF THE MEDICAL CLASS:—

I appear before you upon the present occasion, in obedience to a custom, which demands at the hands of the Faculty of the Atlanta Medical College, an annual salutation to the Students who assemble in their Halls for Instruction.

As the organ of the Faculty, allow me to greet you and to offer in their name, the old and familiar, yet never-to-be-improved term of salutation, *welcome;* to the Halls of the Institution, and the hearts and homes of those I represent. In addition to the custom demanded by courtesy to you, and as an evidence of our appreciation of the confidence which you have manifested by your attendance here, it is also expected that you should receive an Introductory Address, more or less intimately connected with the objects which have prompted your presence on this occasion.— Though the Institution which you have honored with your confidence is only in the fifth year of its existence; and though we would disclaim any intention of presenting either its intrinsic or comparative advantages for patronage, yet, the Faculty have no desire to conceal their hope, that, with the energy which they will assuredly bring to their labor, and the ample appliances which they have accumulated, not only from this country, but from Europe, they will not only be able to deserve the respect and confidence, but advance the real interests of those who avail themselves of the advantages for the acquisition of Medical knowledge, offered in connection with the Atlanta Medical College.

This assemblage of young gentlemen, from distant and different sections of a widely extended country, indicates some object which they conceive demands the sacrifice, in no small degree connected with the separation from home and friends.

We would fain hope, gentlemen, that you have been in some degree prompted by a proper appreciation of the true character of the Science and Art, upon the threshhold of which you are about to enter, and that benevolence and philanthropy have had much to do with your selection of a profession.

He whose aspirations extend no higher than the mere desire for the acquisition of money, we would arrest and point to some other road to fortune; but hoping that higher and more worthy motives have influenced you in the selection of a calling for life, we welcome you to a study which furnishes a pursuit worthy of the most lofty and cultivated minds; and *one* which is not inferior to any other in the whole range of knowledge open to human investigation; and whose advances have been continuous since the commencement of the present civilization. "Striking and brilliant conquests in the domain of discovery have been made, of course, only at long intervals, but its truths have been steadily accumulating," until its embodied certainties have been magnified into the majestic proportions of a great Science.

My object upon the present occasion will be to speak of the *Status of Medicine as a Science and an Art.* It might seem to you unnecessary for me to speak of the worth, dignity and position of that calling which you have selected for your future occupation, upon the ground that you were already sufficiently convinced of its merits and attractions, or you would not have adopted it. But, gentlemen, this is emphatically "the day of free thought, of free investigation, and free speech, in which men do not hesitate to call in question the most hoary as well the most recent fact." Mind of the present day has indeed become reckless of all restraint, and with a freedom of inquiry amounting to licentiousness, hesitates not to intrude upon the most sacred and venerated ground—this is again eminently the age of *toleration*, and according to a late able writer upon the progress of civilization, the spirit of *scepticism* has ever accompanied, during every period of the world, the spirit of *toleration.* The genius of the present age has not, therefore, hesitated to intrude without ceremony into our hitherto sacred places, but dares to question the truth of our most cherished principles; that spirit which hesitates not to doubt the truths of our holy and venerated religion, has not failed to turn an incredulous ear to the time-honored teachings of Medical Science. This being true, it may not be time and labor lost to examine well our foundations, that "we may be able to give a reason for the hope that is in us.

The scepticism of the present day, says, in reference to human progress: "There is no sensible amelioration of man in the essentials of his being, intellectual, moral and physical—in intellect he has stood still, in morals retrograded, in physique degenerated. At our highest point of mental elevation we have not shown ourselves wiser than Plato: when *he* died, his shroud cast a darkness over the world that time has failed to remove. At our most advanced stage of ethical developement, we have sunk below the standard attained by the immediate disciples of ancient moralists—in the harmony and completeness of physical endowment we have fallen from the typical perfection of

Antique Greece, from the matchless models that inspired him who chiseled the Venus or the Apollo. And mark, this is the statement when the highest individuals of one age are compared with the same class of another—a yet more gloomy view is taken when the masses of past and present eras are placed side by side, and this disastrous view reaches its climax with those who maintain progressive degradation, mental and bodily, as characteristic of the age, will be of succeeding ages—*of all time*."* While this view is not true, and man in many particulars occupies a more elevated position than at any period of the world, yet we are compelled to admit that "there is an aspect peculiarly interesting to the Medical Philosopher in which the march of time has effected little improvement in the Physiology of man—in the quality of his emotional nature, and in the extravagances into which this ever and anon betrays him—in the wild love of the marvellous—in the gross worship of the mystic and supernatural. The belief in witchcraft is no longer recognized by law; amiable judges in solemn wig and ermine, no longer burn elderly women because they have been seen riding on broomsticks at lofty altitudes through the air; but the popular faith in witchery in many parts of the civilized world, is as implicit as it was centuries ago." What right have we to "shrug our shoulders with disdain at the benighted condition of the credulous old Roman, who firmly believed that the Augur could foretell any amount of future events, by peering into the bowels of an animal?" Have not the *miracles* of designing men in modern times, taken the place of the witchcraft and oracular wisdom of former days? "Are there not people who believe that others can read ad libitum with their epigastria? Is there not a body of men," and especially *silly women* (I say it, with all that profound respect and admiration which I have for true and sensible female character,) "otherwise apparently sane, who laughing old Euclid to scorn, act not only as if a part were greater than the whole, but as if, the smaller that part be made, the greater the

*Passing Glance at Human Progress, by Walshe.

superiority to the whole that furnished it? A grain is for potency, *nil*, but the decillionth part of that grain is, in inexperienced hands, dangerously powerful. Yet more, have not grave Divines written treatises denouncing the impiety of those whose faith fails to raise them to the belief point in table turning and to the knowledge of its true mechanism, namely, the agency of Satan himself. And as an improvement on the spiritual vision of the timid lexicographer, Dr. Johnson, who believed in the Cocklane Ghost, have not the means of holding converse with the whole race of departed Spirits been provided for the inhabitants of earth at large? To the somewhat intellectual, but prominently moral and emotional weakness illustrated in various phases by the different examples adduced, may be mainly traced the success, so far as the public are concerned, of the heresies and charlatanisms that every now and then arise to interfere, *pro tanto*, with the progress of true Medical Science. The reign of each delusion, it is true, is short; it lasts till each having grown familiar, has ceased to be wonderful—has lost its claims to marvel worship. But so long as the same physiological and effective element in the constitution of man exists uncorrected, fresh fallacies will continue to replace old ones, scientifically refuted and popularly worn out." In reference to the question, however, whether there is a human progress, I would reply that in this connection, as ever, truth lies between—that *there is a progress* in many essential particulars. While it is true that there has been very little change in those great dogmas which have been given us as a moral system—while "there has been no change in the great essentials of morals, to do good to others; to sacrifice for their benefit our own wishes—to love our neighbor as ourself—to forgive our enemies—to restrain our passions—to honor our parents—to respect those in authority—not one jot or tittle having been added to them by all the sermons, homilies and text books which moralists and theologians have been able to produce," and while it is also true, as before remarked, that the march of time has effected but little change in man's emotional nature, rendering him constantly liable to be led astray, and making him still an easy prey to deception and delusion, from his love of the supernatural and marvellous, yet *there is a progress.*

"If we contrast this stationary aspect of moral truths with the progressive aspect of intellectual truths, the difference is indeed astonishing. All the great moral systems which have exercised much influence, have been fundamentally the same—all the great intellectual systems have been fundamentally different. In reference to our moral conduct, there is not a single principle now known to the most cultivated people on the earth, which was not likewise known to the ancients. In reference to the conduct of our intellect, the moderns have not only made the most important additions to every department of knowledge that the Ancients have ever attempted to study, but besides this, they have upturned and revolutionized the old methods of inquiry—they have *consolidated into one great scheme, all those resources of induction* which Aristotle alone dimly perceived; and they have created Sciences, the faintest idea of which never entered the mind of the boldest thinker antiquity produced."*

While, therefore I profess, to be a great admirer of antiquity, I would not be solely occupied in venerating past ages, but learn to respect the present, and hope for the future. While I would urge you to reject every *novelty* not based upon *reason*, I would as warmly urge you to adopt every *truth* which can be established by a rigid system of *induction*, whether it be the offspring of to-day, or a part of the wisdom bequeathed by your fathers. In either point of view, gentlemen, our calling has nothing to fear from investigation, but everything to encourage the lover of humanity or the student of science.

As you are aware, history cannot determine its antiquity; we have, however, good reason to conclude that, "among the civilizations which may have flourished before the commencement of Egyptian and Indian records, and then dis-

*Bnckle's History of Civilization.

appeared from the earth like the extinct Mastodons and Mammoths; there is little doubt that systems of medicine more or less complete, perished at the same time with other evidences of refinement and cultivation. Whenever, since then, the human race has made any intellectual progress, medicine has always received its due share of attention. The greatest and most ancient of epic poets that history knows, does not think it beneath the dignity of his verse, to do homage to the worth and calling of the physician. Throughout the classic periods, medicine was cultivated by the most active and intelligent of the times, and when the darkness of a Gothic night settled down over the face of Europe, it was preserved from destruction in the literature of the studious and sober Arabs. Since then, no science has had brighter ornaments or more devoted followers.

Following Hippocrates and Galen, Celsus and Pliny, and Avicenna, have come Morgagni and Malpighi, Spallanzani and Vesalius, Ambrose Pare, Harvey, and Hunter, and Boerrhave and Bichat and Læennec. Do you think such minds as these can have labored in turn for two thousand years in the field of Medical research, without leaving us a legacy worth inheriting? The subject that occupied them, enriched as it is by their successive accumulations, can not certainly be unworthy of cultivation by us." The achievements of the medical men of the past have reared a temple for Science and Truth not surpassed by the results of any human labor.

> "See where aloft its hoary forehead rears,
> The towering pride of twice a thousand years;
> Far, far below the vast incumbent pile
> Sleeps the gray rock from Art's Egean isle;
> Its massive courses circling as they rise,
> Swell from the waves to mingle with the skies.
> There every quarry lends its marble spoil,
> And clustering ages blend their common toil;
> The Greek, the Roman, reared its ancient walls,
> The silent Arab arched its mystic halls;
> In that fair niche by countless billows laved,
> Trace the deep lines that Sydenham engraved;
> On yon broad front that breasts the changing swell,
> Mark where the ponderous sledge of Hunter fell;
> By that square buttress look where Louis stands,
> The stone yet warm from his uplifted hands;
> And say, O Science, shall thy life blood freeze,
> When fluttering folly flaps on walls like these."

And, Gentlemen, "the work still goes bravely on," while there may be many drones in the ranks, the medical men of the present are not resting upon the labors and achievements of their predecessors, but are actively engaged in performing their part in this great work. The men of the present are not inferior to those of the past, nor less entitled to the respect and confidence of the world, than they ever were. If there is more scepticism in reference to the claims of legitimate medicine, it is, to a considerable extent, the result of the causes to which I have already adverted, and largely, in addition, the other extreme to that blind credulity with which the world received the medical dogmas of former ages. Only another swing upon the opposite side of that golden medium where truth is ever found—another cause for incredulity upon the part of narrow and contracted minds, is found in the very progressive character of the science itself; with their eyes shut to the analogy found in other science, the exploded theories of the past furnish to them an argument against the truth of the present.

Again, to the superficial observer, this would seem to be an age eminently prolific of empiricism and charlatanry, and it is true that we have had almost innumerable forms and systems of quackery,(so called medicine)—Homœopathy, Hydropathy,Thompsonianism, Eclecticism,Indian Medicine, Chrono-Thermalism, Natural Bone Setters and Female Physicians, with the almost interminable list of nostrums and vagrant quacks, the most of which have furnished a representative in making up the remarkably varied population with which you will find yourselves surrounded during your sojourn here. But, gentlemen, these or some other forms of delusion, imposition and heresy, have always existed, must, and always will, be found in the world ; so long at least, as we have venality, ignorance and credulity. It is, however, the peculiar tendencies and associations of the quackery of the present day, that makes it a matter at all worthy our attention ; not so much then, as medical men, as the lovers of virtue and a sound morality, and in this point of view we could wish it could receive the attention of every intelligent and moral mind in the land, believing as we do, that a consideration of the subject would be sufficient to induce every virtuous individual to turn with disgust from the labors and teachings of these *new lights* in morals, as well as in medicine.

This is not the occasion, nor have I the time to go into the details of this subject, but assert upon reliable authority, that in the northern portion of the United States, which may be regarded as the very hot-bed of the multiplied forms of quackery, we find those who are the advocates of Spiritualism, Mesmerism, Fourierism and Free-Love, *the special disciples* of the Hydropathic and Homœopathic delusions, and the promulgators of the doctrine, that a marriage becoming distasteful to either of the parties, it is the duty of that party to dissolve the connection and enter upon a new one with some more *congenial* spirit—and from a pseudo physiological stand point, an attack is made, not on hasty, ill-considered or unnatural marriages, but on the institution itself—on the Bible, as recommending it, and on the legitimate profession of medicine, as the well known opponent of licentiousness. In the so called medical works of these writers, may be found the impersonation of all heresy and schism; of hatred to that medical science and profession which it cannot bend to its doctrines, an admixture of Water Cure, Homœopathy and Spiritualism—a monstrous and spurious physiology, whose insane teachings lead its followers over the ruins of the social fabric, morality and religion. A debasing sensualism, which, in the name of friendship to the gentler sex, would sink them to the lowest degree of degradation. Such are the doctrines and teachings to be found in the therapeutics of Hahneman and Preissnitz, respectively the great heads of the most fashionable of the medical follies of the day—Homœopathy and Hydropathy; the text-books of the former being filled with stupid transcendentalisms, which befog and prepare the mind for that farrago of nonsense to be found in the writings of the spiritualists and communists, that balderdash about "higher harmonies," "marriage of the affinities" and "passional attraction." The very nosology of Hahneman, making vice a disease, and a disposition to lie or steal, or murder, to be cured by thirtieth potencies, and in this point of view making as it has been said, "the practice of Homœopathy, peculiarly appropriate to penitentiaries and jails."

But the great point which stands prominently forth in this matter, is that the adoption of such theories, implies at once a complete upsetting of all power to discriminate between right and wrong, and the practical exhibition of such a bond of affinity between all the various forms of quackery, and those of religious and social infidelity, that we find the same class of minds attracted by both—those who daintily tamper with one, being exceedingly liable to be entrapped by the other—the highly elevated, and truly conservative position of the medical profession proper, being most forcibly illustrated by the fact, that all the more prominent "*Medical Reformers*" of the present day, are persons of low morality.

This is a portraiture drawn by those* who are in the very midst of these corrupting innovations of the day, the clear tendencies of which are to sap the foundations of our social structure; and the fountains from which these streams of moral poison constantly issue, are the presses of that infamous abolition sheet, the New York *Tribune*, and that of Fowler & Co., New York, whose bare names upon any publication should seal it to every lover of truth and right.

But, Gentlemen, in a physiological point of view, these follies seem to be *required* by a particular class of minds, and may thus be regarded as one of the *necessities* of the world, and it is therefore a matter of but slight consequence with us whether this or that form of medical folly be in the ascendant at a particular period. Be not then discouraged, for you may rest assured that the mass of the world are ever disposed to the side of truth, and that "the remaining fraction, more or less, consists partly of minds so constituted that they require the marvellous as a portion of their necessary food, and partly of unfortunate beings suffering the

*Buffalo Medical Journal and American Medical Gazette.

inevitable lot of humanity, who, having expected too much, and failed to obtain relief from the ordinary resources of medicine, seek for temporary encouragement in the dishonest assurances of any who will promise to cure; the first class, is the dog in the fable catching at shadows; the last is the drowning man catching at straws."

To the medical philosopher, there is another interesting view of this subject which is singularly consoling. It is in the highest degree important to ascertain whether or not there exists a regularity in the entire moral conduct of a given society, and this is precisely (as remarked by a late able writer upon civilization) one of those questions for the decision of which *statistics* supply us with materials of immense value. An immense mass of statistics have been gathered by European governments in reference to crime, the conclusions from which throw, in my judgment, a flood of light upon the whole moral and mental nature of man. The great advance made by the statisticians consists in applying to these enquiries the doctrine of averages, which no one thought of doing before the eighteenth century.

Whoever has become acquainted with the results of investigations which have been made in reference to the *recurrence* of mental and moral phenomena, and the regularity with which they occur and succeed each other, cannot, I think, regard it as unreasonable to conclude that we are merely in the infancy of development in reference to the practical working of this great world, and must anticipate constant additions to the important demonstrations which have been made by *statistics*, in regard to the uniformity in human affairs.

Buckle remarks, and sustains his view by unquestionable data, "that murder is committed with as much regularity, and bears as uniform a relation to certain known circumstances, as do the movements of the tides and the rotations of the seasons. M. Quetelet who has spent his life in collecting and methodizing the statistics of different countries, states, as the result of his laborious researches, that in every thing which concerns crime, the same numbers reoccur with a constancy which cannot be mistaken ; and that this is the case even with those crimes which seem quite independent of human foresight, such, for instance, as murders, which are generally committed after quarrels, arising from circumstances apparently casual. Nevertheless we know from experience, that every year, there not only take place nearly the mase number of murders, but that even the instruments by which they are committed are employed in the same proportion." This was the language used in 1835, by confessedly the first statistician in Europe, and every subsequent investigation has confirmed its accuracy.

Again, notwithstanding the peculiarities of that most singular crime, suicide, rendering it, we would suppose, almost impossible to trace it to those general causes by which it is produced, all the evidence we possess points to one great conclusion, that whether as stated by an investigator upon this subject, in a given state of society, a certain number of persons *must* put an end to their lives, the general law is—"In the different countries for which we have returns, we find year by year the same proportion of persons putting an end to their own existence, so that after making allowance for the impossibility of collecting complete evidence, we are able to predict within a very small limit of error, the number of voluntary deaths for each ensuing period, supposing of course, that the social circumstances do not undergo any marked change. Even in London, notwithstanding the vicissitudes incidental to the largest and most luxurious capitol in the world, we find a regularity greater than could be expected by the most sanguine believer in social laws ; since political and mercantile excitement and the misery produced by the high price of food are all causes of suicide, and are all constantly varying. This is only a part of the evidence we possess of the regularity of the actions of men, and we must recollect that the facts stated are not the arbitrary deductions from a partial selection from particular facts, but that it is a generalization from an exhaustive statement of criminal statistics, consisting of many millions of observations extending over countries in different grades of civilization, with different laws, different opinions, morals and habits. The facts then, in regard to crime, may be regarded as more clearly attested than any other in the moral history of man.

"Nor is it merely the crimes of men which are marked by this uniformity of sequence (and what may appear very strange and perhaps incredible to the more romantic part of my audience, and the announcement of which, may be considered somewhat ungallant upon my part,) even the number of marriages annually contracted is determined, not by the disposition and wishes of individuals, but by large general facts, over which individuals can exercise no authority. It is now known that marriages bear a fixed and definite relation to the price of corn; and in England the experience of a century has proved that instead of having any connexion with personal feelings, they are simply regulated by the average earnings of the great mass of the people, so that this immense social and religious institution is not only swayed, but is completely controlled by the price of food, and the rate of wages. In other cases uniformity has been detected, though the causes of the uniformity are still unknown. Thus to give a curious instance, we are now able to prove that the aberrations of memory are marked by this general character of necessary and invariable order. The Post Offices of London and Paris have lately published returns of the number of letters which the writers, through forgetfulness, omitted to direct and making allowance for the difference in circumstances; the returns are year after year copies of each other. Year after year the same proportion of letter writers forget this simple act; so that for each successive period we can actually foretell the number of persons whose memory will fail them in regard to this trifling, and as it might appear, *accidental* occurrence."

Who shall limit then (after these achievements,) the results to be derived from statistics, a branch of knowledge, which though still in its infancy, has already thrown more light on the study of human nature than all the sciences combined.

"To those who have a steady conception of the regularity of events, and have firmly seized the great truth, that the actions of men being guided by their antecedents, are in reality never inconsistent, but however capricious they may appear, only form part of one vast scheme of universal (and providential) order, of which we, in the present state of our knowledge, can barely see the outline; to those who understand this, which is at once the key and the basis of history," the facts just adduced will justify me in the suggestion that the impositions and delusions of the present day, in regard to medicine, are only other examples of the operation of a fixed law, which has produced in various periods of the world's history, a thousand and one vagaries, none of which have been too absurd to find adoption—only a recurring peculiarity of human action which in some form or another, is each year finding its duplicate. To those who might object to the theological view, which might seem to be presented, by a superficial examination of this subject, all I have to say is, that we are informed by our direct consciousness of the existence of feelings and ideas and of the control by the *will* of our thoughts and actions, which is to us the *most certain* of all realities. We can only say that under certain recurring circumstances, the honesty of some and the common sense of others, is brought into a state of suspension or subjection. And under this head we may range the wild but transient vagaries of *religious enthusiasts* in all ages; the ecstatic revelations of Catholic and Protestant visionaries; the preaching epidemic among the Huguenots in France, afterwards in Sweden, and more recently during the excitement of Millerism and Spiritualism in our own country; the dancing mania of the middle ages, which exhibited men and women dancing in a circle, shrieking and calling wildly on John the Baptist, and at last, as if seized with epilepsy, tumbling upon the ground where they desired to be trodden upon and kicked by the bystanders, who it is said, did not, (as they should not,) hesitate to comply with perfect good will. In the olden time, the ladies of Miletus, in a fit of melancholy for the absence of their husbands and lovers, resolved to hang themselves, and as in all fashionable amusements, vied with each other in the alacrity with which they carried on their work of self-destruction; and a still later manifestation of mental delusion more directly connected with our science, was exhibited in the annual visit to the Chapel of St. Vitus, of women laboring under some nervous derangement where they were said to dance violently and unremittingly from morning until night, or fall from exhaustion, by which they imagined themselves cured of some real or fancied disease.

But, not to prolong this subject, which has already grown tedious, allow me to assure you that nothing is to be feared from the aberrations of the world in reference to our Science. If Pseudo Medicine, in its various forms of *pathies* and *isms*, is advancing at one point, it is receding elsewhere; if progressive to-day, it is the reverse to-morrow, and is only obeying the law of its nature: "thus far shalt thou go, and no farther." But, gentlemen, whatever may be the fact in reference to other subjects, our Science is neither stationary or uncertain. While I would not hold out the idea of a *perfect* Science or Art, I have no hesitation in asserting that, excepting Mathematics and pursuits resting strictly upon it, no calling of man surpasses Medicine in the certainty of its opinions, and none, most certainly, equals it in its positive, and increasing blessings to society. It is eminently progressive, and its record is equally honorable in this particular, with that of any other Science or Art known to the world. "If we examine the great eras in civilization, Medicine will be found to have progressed as rapidly as the physical Sciences generally. The discoveries of Columbus and successive navigators were not earlier or more important in Geography, than those of Mondoni, Beranger, Vesalius, and Sylvius, in Anatomy. Copernicus did not earlier conceive the errors of the Ptolemaic Astronomy than Servetus and Cesalpine the errors of Galenic Physiology; and Galileo who demonstrated the movements of the earth and planets around the sun, was a cotemporary with Harvey who demonstrated the circulation of the blood. The universal law of Newton for the solar system, was not greatly in advance of that of Haller, of the laws and special forces of life. If the great philosopher established that the force manifested in the fall of an apple to earth, is the same as that which keeps the planets in their orbits, so the pathologist has shown that the laws of inflammation in the deep-seated and vital organs, are identical with those that are *seen* in the smallest inflammatory point on the skin."* "While there has been but little progress for a long period in Theology, Law, Belles-Lettres, the Fine Arts, Architecture, Politics, Mathematics or Metaphysics, Medicine has kept pace with Mechanics, the Chemical and Physical Sciences, the most rapid progress in which, is characteristic of this stirring age."— From general ignorance of all knowledge that elevates mankind above the beasts of the field, found as well among the rich as the poor, in the house of luxury as in the abode of destitution, a portion of the world know nothing of the sources from which have been derived many of the blessings which they enjoy; and even among those who are intelligent upon other matters, from want of attention to the subject, there is a failure to acknowledge their obligations to the profession of Medicine: notwithstanding this, however, we have the encouraging fact that the mass of the civilized world are to a greater or less extent responsive to the claims of our Art. While it is true, as already stated, that certain portions of mankind adopt, in succession, the legion of follies which assume the garb of Medical Science, and are thus recreant to their obligations, and practically deny its existence, it is also true that the honest and intelligent investigator of history and truth can not fail to admit the argument furnished by *statistics*, which demonstrates beyond all cavil, the inestimable benefits conferred by the labors of Medical men, upon suffering humanity.

---

*Renouard's History of Medicine, by Comegys.

These *statistics* which we have already used in the attempt to establish another proposition, show, that human life has been greatly lengthened in the last hundred years, indeed, more than twenty-five per cent. in the last 75 years, and the duration of the treatment of disease, has been lessened one third; and Medicine cannot be deprived of her right to the honor of these results, by arguments drawn from a diminution of mortality in consequence of a cessation of war or improvements in the Hygenic conditions of mankind; for besides the latter being distinctly traceable to the labors of Medical men, "the *statistics* connected with Parisian Hospitals show that while in 1805 one died in seven, who were admitted, now only one dies in 12, thus showing that our Science has increased in its ability to save life, in the same order of diseases and in the same buildings, 71 per cent. in a period of 50 years;" or in other words, in the 80,000 who annually pass through those wards, we have a saving of 500 human beings, with the period of their suffering greatly lessened. In England the term of human life has been greatly lengthened; the returns of the Registrar General of England show a steady and notable decrease in the rate of mortality—the difference, it is said, between London in the 17th and London in the 19th century, being as great as between London in ordinary years and the same city in the Cholera. In France the duration of life has increased in the ratio of 52 days for each year, during a period of 66 years, for which we have the *statistics*, although that revolutionary and warlike nation shed seas of blood, not only in her Cities, but upon every battle-field of Europe." In Surgical practice, the saving of life at present exceeds by more than one third the results of operations at the commencement of the present century. In the practice of that branch of Medical Science, around which clusters such interesting associations and gloomy apprehensions, which make the profoundest appeals to the sympathy of the Medical man, and which is so intimately connected with our *earliest* appearance in the world, "it is stated by Dr. Merriman, that one hundred and fifty years ago, one in forty died, and at the close of his tables in 1828, only one in one hundred and seven died; and at this time (in the hands of scientific men) perhaps not one in 250 dies." And, gentlemen, I have the great pleasure of stating that the Hospital practice of our own country exhibits the same gratifying success in the treatment of disease, the statistics of the Hospitals of our large cities showing alm( st precisely similar results to those furnished from Europe. "In short, whether we examine the reports of the Registrar General, of England, the data of the Carlisle and Northampton life tables; the *statistics* of the Bureau Centrale, of Paris, or the publications of the Great Hospitals of our own country, the same results are presented,"* and thus Medicine claims a position in the calculations of the political economist; and it is not presumption to say that the enlightened physician rises to the highest rank among practical statesmen. "A philosopher of the last century actually maintained that a period of man's history would eventually arrive when death would be the result of extraordinary accident, or of slow destruction of the vital forces (itself becoming a slower and slower process as the world advanced,) and the duration of life would extend beyond any now assignable limit. Whether human existence will ever reach any such term of protraction as is signified in this hypothesis, may reasonably be doubted, but that in countries of high civilization, the mean length of life has notably increased within the period of mortuary records, *is certain;* and that this increased longevity is due to our Science, is unquestionably proved by evidence contained in *statistical* reports." Indeed if we had time, and this were the occasion, it would be an easy task to enter the field of *special* diseases, and show in the most conclusive and palpable manner, the achievements of Science. It would be an easy task to show that, by a more thorough knowledge of Physiology and Pathology, our means of Diagnosis have been so much improved that there are but

---

*Comegys.

few derangements of the human system which cannot be detected with as much accuracy as the machinist can determine the point and nature of any disturbance in a complicated mechanism. Epidemics have been shorn of their terrors; Small Pox and Scurvy which have left in their train desolation and ruin, are comparatively unknown, a result most distinctly and palpably traceable directly to our Science. Even a ray of light has been thrown along that dark and gloomy path which has been considered as pre-eminently *the way of death.* There is now even hope that man may be rescued from the jaws of that fell destroyer, *Consumption,* than which, (in our large cities) no malady brings the pawn-broker so many warm garments from devoted, self-sacrificing, and shivering women, or fills his drawers with so many rings of plighted love, to buy the last delicacies to sustain lingering life. There is now even hope that humanity may be rescued from that disease which is so full of bitter memories to the physician, for it tells him of imploring looks, answered by forced smiles, with a heavy heart; of blasted hopes and arms folded in despair; of the noblest of kindred and friends struck down in the brightest period of life; of the ruthless entrance of death, and the tearing away of the gentlest lambs of every flock; of those who though most ready to die, were most worthy to live.*

And then, again, gentlemen, our science has not confined itself to the relief of mere physical disease; "through its instrumentality the sufferings of the outcast have been alleviated and relieved, the wandering intellect has been allured from its eccentric orbit back to its legitimate sphere; the breaking heart has been bound up by the hands of kindness and skill, and taught to thrill again to the magic touch of love and friendship; and the shattered link of many a sorrowing family has been restored in all its pristine strength and brightness, to the eternal gratitude of those who mourned it as lost forever. Through its benign instrumentality, the prison-house has been entered and the cankering fetter stricken from the limbs of him, whose mental ruin was his

*Corson.

only crime. At its bidding, the loathsome dens, to which affection, in the blindness and infatuation of a false philosophy had confined the objects of its warmest solicitude, gave up their victims to the merciful dispensations of a more judicious philanthropy. Under its gentle and elevating influence, *a new era* has dawned upon the destiny of those unfortunate beings, whom heaven has cursed with *insanity*; before whose radiant beams the darkness and gloom of many a noble mind and generous heart have been scattered as the mists of the morning, by the rising sun." The towering walls, enlightened philanthropy and genial appliances, devoted to the insane, are perhaps the noblest trophies with which modern medicine and modern civilization are adorned.

Then, Gentlemen, be re-assured of the value and importance of the vocation you have selected, and congratulate yourselves that you have embraced a profession which is occupied with questions so varied, and interests so vast; but I dare not fail to tell you that its obligations and responsibilities are serious and fearful in proportion to the dignity of its purposes and the lofty character of the objects it proposes to accomplish. You are to be no less the conservators of the public health, (sanitary and hygienic science being the exclusive creation of your profession,) than the healer of its maladies when the subjects of disease.—Remember then, you may with pride and hope (if determined never to enter upon it, unqualified to discharge its high duties,) that you have adopted a profession which is the representative of neither *Allopathy* (a term your defamers have applied,) or any other pathy or ism, but elevated above mere pills and potions and powders, stands forth as the practical part of the great science of medicine—the accumulated truths of thousands of years, which, "while it cares for the individual, concerns itself also with the masses—with the species—aims at indirectly purifying the morals, vivifying the intellect and lengthening the mean span of existence of mankind at large." But, gentlemen, this view would not be complete, if I did not allude to what is said by some *in*, and others out of, the profession, who will admit the progress of medical science; that the *medical men* of the present day are the unworthy representatives of worthy sires; indeed we find constant comparisons instituted between the profession of our day and that of the past, unfavorable to the former. It is stated that the art of modern medicine fails to come up to the demand naturally excited upon the part of the world, by the large pretensions of the science upon which it professes to be based. To this proposition, we cannot conceive any more conclusive reply than is furnished in our records, which (without attempting details,) will show that there never has been a period in which medical science has advanced more rapidly than in the last few years, and there never was a period at which medicine contained so large an accumulation of undisputed facts—at which there were so few theories not established by a rigid system of induction, or at which there was so much unanimity in reference to the principles of physiology and pathology, (the foundations of medicine,) and never so much unity of view, as to the best modes of accomplishing the great objects of our science, the preservation of the bodies and minds of our race, and never a period (as has already been most conclusively shown,) at which the practical results exhibited by the efforts of those who were engaged in the active labors of the profession were so eminently satisfactory. With what show of justice or reason, therefore, can such a reflection be cast upon those who now represent *a still progressive work?* But it is said, again, however true may be the inference drawn from such a statement of facts, so far as Europe is concerned, where those who seek a place in the profession are required to spend more time in preparation before they are allowed to assume its responsible duties, it must be admitted that American medicine is a degenerate offshoot from its European parent. It has even become a favorite object with some who claim a place in our ranks, to degrade the American medical profession, in the estimation of the world, and especially to decry what is called in derision the "American System of Medical Education." Now, gentlemen, I am not here upon this occasion as the special champion of the institution which you have honored with your presence, or of any other medical college. Nor am I here to defend and endorse *as perfect*, the plan of educating medical men in the United States, but I am free to confess that I am heartily sick of, and prepared to denounce, as false, the stereotyped cry of the inferiority of the medical profession of the United States, as compared with that of Europe. It is high time in my judgment, that the supercilious and superficial traducers of American medicine, in or out of the profession, should be met by the facts. I would not wish to discourage any effort upon your part to master all the great truths connected with our science, but rather to encourage you to the most indefatigable efforts to fit yourselves for the faithful discharge of the solemn obligations resting upon all those who assume the responsible position of physician. But I consider myself fully authorized in asserting that the regular medical men of the United States are offering an amount of science and skill far exceeding their appreciation upon the part of the public, and furnishing a standard of qualification far higher than the people deserve, and fully equal to the demand—the highest order of science and skill in every department of our comprehensive profession, being readily and conveniently commanded by every community that has any proper conception of the remuneration that should be awarded to the self-sacrifice and toil, which you may be assured, is ever inseparably connected with eminence in medicine.

It is yet true, that we have in our ranks, every where, and it is admitted with sorrow and shame, those who, from ignorance and vice are a disgrace to our calling—those who are better acquainted with cards, billiards and dice, than with Anatomy, Physiology and Materia Medica; those who find the saloons of vice and infamy their congenial spheres; *some* who, as Zimmerman says of Doctors in Chili, "*Blow* about their patients and think *they know enough* when they know *how to blow*;" others whose only skill is in covering up their ignorance, and cunningly *thrusting* a more conscientious and laborious professional brother, by hinting that it is not well to be *too scientific*, and still others who pursue the many devious by-ways suggested by a corrupt human nature. But, gentlemen, these are the foul fungi clinging to the surface of the lofty oak, the barnacles upon a noble ship, the scullions and scavengers of the Medical Profession, whose counterpart is found in all the professions; in the pettifogger of the Law, and in the ignorant and often unprincipled expounder of Sacred Truths.

I would then say in reference to any supposed inferiority of American Medicine and American Medical men to those of Europe, which I do not admit, that whatever defects belong to the results of our system of Medical Education, attach equally to your whole plan of education; to your primary school, to your Academy and Seminary, (if there be any such thing); to your college, to your law and theological schools; to the education of both sexes for all the departments of life. "Cheapness of education and a corresponding adaptation of time are found indispensable to the general condition of society. He who, in America, is aiming at the Profession of Medicine, and has met with no obstruction to his rapid advancement, in his primary, academic or collegiate course, will not be arrested by exactions found in no other department of education and preparation for active life, but finding almost innumerable avenues open to wealth and distinction, demands a means of ingress to the Medical Profession, corresponding with that found connected with other callings. Deny this, and we divert those who are disposed to enter the Medical Profession, into one of the various walks of irregular and empirical Medicine."*

But this has not been found to be incompatible with eminence in Medicine—indeed we learn from a late reviewer of Medical Education in Great Britain, (which may be taken as a fair representative of Europe,) as exhibited in an elaborate article in the Westminister Review, that there is

*Professor Martyn Paine, of New York.

great injustice in supposing that the American Medical Profession can be benefitted by any usage or example supplied by the Profession in Great Britain ; but on the contrary, it is shown that there is no advantage in the comparison to the European Medical man, in either mind or attainments. And we heartily endorse the conclusion to which he comes in regard to the real merit and true position of the representatives of American Medicine at the present day, and what no one can successfully gainsay, that as a class, neither the American Lawyer or Divine is more intelligent, more industrious or better educated or more cultivated in manners, or better qualified for his vocation, when he enters upon its duties, than the graduate fresh from our Medical Colleges; and in this parallel is only included the Divine and the Lawyer who have enjoyed the opportunities of Theological and Law School education. "And without intending to impertinently claim for the Medical Profession any ascendancy in another particular above the clerical and legal ranks, we are entitled to point with pride at the noble bearing of the mass of American Physicians who, notwithstanding their great numerical ratio, very rarely, almost never, scandalize society by the commission of crime; but on the contrary, are especially distinguished for moral deportment, which is spoken in no arrogant tone or spirit of exultation, but simply for the honest contemplation of all those who have failed to do justice to American Physicians.*

I can not, therefore, consider it inappropriate to the subject or the occasion, to declare upon the highest authority, the testimony being based upon a practical familiarity with Europe—and after a somewhat laborious investigation of this question, that the American pupils in attendance upon Foreign Schools of Medicine, are in no ordinary degree superior to the classes with which they are associated, and that the mass of physicians in Europe are in no respect superior to the mass in the United States, but on the con-

---

*N. Y. Medical Press, containing an able and conclusive defense of American Medicine.

trary, in their treatment of disease, it is fearlessly repeated that they are decidedly inferior.*

In the rapid march of medical improvement, then, gentlemen, the records of our science will show that no inconsiderable part is due to the labors and contributions of your own countrymen. A large proportion of those who enter the profession, belong to the most refined and educated classes in their respective communities, and though "they are rather remarkable for smoking and chewing tobacco," as a whole, do credit to themselves, to their instructors, and to their chosen profession; and "any man is unworthy his birthright, who does not discountenance the wordy tirade poured out so abundantly in certain quarters, in disparagement of the education and standing of the great body of American physicians. It is high time then, that Americans should cease to decry the efforts of their own medical scholars, and the attempt to degrade the whole profession of their own country, and sacrifice their own medical literature for what is conceded to be the inferior medical literature of Great Britain." I feel, then, gentlemen, from an investigation of this subject, (a mere glance at which alone I have been able to present,) that while I yield to no one in the estimate I place upon the eminent medical men of Europe, the mass of medical men of the United States are *superior* to those of Europe in practical knowledge of, and skill in the treatment of disease—in independence and vigor of thought and action, and in all the elements that constitute a higher order of man, and that their stand-point for future improvement is far more elevated than that of the physicians of the old world. And, gentlemen, this is not at all strange to the close observer of man upon the earth.—"Asia, Europe and North America are the three grand stages of humanity in its march through the ages. Asia is the cradle where man passed his infancy under the authority of law, and where he learned his dependence upon a sovereign master. Europe is the school where his youth was trained, where he waxed in strength and knowledge, grew to man-

*Caldwell.

hood, and learned at once his liberty and his responsibility. America is the theatre of his activity during the period of manhood; the land where he applies and practices all he has learned; brings into action all the forces he has acquired, and where he is still to learn that the entire development of his being and his own happiness are possible only by willing obedience to the laws of his maker."

"Thus lives and prospers under the protection of the divine husbandman, the great tree of humanity, which is to overshadow the whole earth. It germinates and sends up its strong trunk in the ancient land of Asia. Grafted with a noble stalk it shoots out new branches, it blossoms in Europe. In America only, it seems destined to bear all its fruits. In these three we behold at once, as in a vast picture, *the past, the present and the future.*"* But, gentlemen, if this be true, it is a solemn fact, "for unto whom much is given, of such, much will be required." We have duties to perform, commensurate with the gifts with which we have been endowed, and let me urge you to be responsive to the claims which your high vocation imposes upon you. In conclusion, gentlemen, of what I know, has been a tedious, and I fear, a desultory glance at the present position of the science and art which you have adopted, and to acquire a knowledge of which you are here, permit me to hope, that by your labors you will hasten on the march of practical improvement, which is not more remarkable in any other science or art, than in modern medicine, *in which*, a severe and accurate process of reasoning has superceded the construction of ingenious theories based on brilliant conceptions, and relieved us from the influence of the imaginings of the ancient or modern dreamer—the foggy theories of past ages having been dissipated under the influence of the rigid modern philosophy, bringing us, *at least*, we hope, into the *dawn* of the full light of scientific truth.

The period having arrived when "the flowers appear on the earth, and the singing of birds and the voice of the turtle is heard," and when the husbandman is abroad in the land, committing the seed to the soil, and with every energy on the alert, in the preparation for its future culture, that he may gather in

*Guyot's Earth and Man.

full stores hereafter; permit me to unite with the interesting and exciting associations of this delightful season, in persuading you to zeal and untiring perseverance in the pursuit of knowledge, that when "the harvest is past, and the summer is ended" no one of you may be found with garners unfilled.

And, finally, gentlemen, indulging the hope, that I may have, in some degree, contributed to your better understanding, and higher appreciation of the true character and position of medicine, I leave with you as a concluding sentiment in reference to our noble profession: "If you look to its *antiquity*, it is *most ancient*—if to its *dignity*, it is *most honorable*—if to its *scope*, it *embraces a world in its arms.*"

---

